# Assimilating MODIS data-derived minimum input data set and water stress factors into CERES-Maize model improves regional corn yield predictions

**DOI:** 10.1371/journal.pone.0211874

**Published:** 2019-02-25

**Authors:** Ho-Young Ban, Joong-Bae Ahn, Byun-Woo Lee

**Affiliations:** 1 Department of Plant Science, College of Agriculture and Life Sciences, Seoul National University, Seoul, Republic of Korea; 2 Research Institute of Agriculture and Life Sciences, Seoul National University, Seoul, Republic of Korea; 3 Department of Earth Environmental System, Pusan National University, Busan, Republic of Korea; College of Agricultural Sciences, UNITED STATES

## Abstract

Crop growth models and remote sensing are useful tools for predicting crop growth and yield, but each tool has inherent drawbacks when predicting crop growth and yield at a regional scale. To improve the accuracy and precision of regional corn yield predictions, a simple approach for assimilating Moderate Resolution Imaging Spectroradiometer (MODIS) products into a crop growth model was developed, and regional yield prediction performance was evaluated in a major corn-producing state, Illinois, USA. Corn growth and yield were simulated for each grid using the Crop Environment Resource Synthesis (CERES)-Maize model with minimum inputs comprising planting date, fertilizer amount, genetic coefficients, soil, and weather data. Planting date was estimated using a phenology model with a leaf area duration (LAD)-logistic function that describes the seasonal evolution of MODIS-derived leaf area index (LAI). Genetic coefficients of the corn cultivar were determined to be the genetic coefficients of the maturity group [included in Decision Support System for Agrotechnology Transfer (DSSAT) 4.6], which shows the minimum difference between the maximum LAI derived from the LAD-logistic function and that simulated by the CERES-Maize model. In addition, the daily water stress factors were estimated from the ratio between daily leaf area/weight growth rates estimated from the LAD-logistic function and that simulated by the CERES-Maize model under the rain-fed and auto-irrigation conditions. The additional assimilation of MODIS data-derived water stress factors and LAI under the auto-irrigation condition showed the highest prediction accuracy and precision for the yearly corn yield prediction (R^2^ is 0.78 and the root mean square error is 0.75 t ha^-1^). The present strategy for assimilating MODIS data into a crop growth model using minimum inputs was successful for predicting regional yields, and it should be examined for spatial portability to diverse agro-climatic and agro-technology regions.

## Introduction

Monitoring crop growth and predicting yield are essential for proper crop management, agricultural operation improvement, and food-security policy decision making [1–2]. Crop growth modeling and remote sensing have been useful tools for monitoring and predicting crop growth and yield [3–4]. However, each tool has inherent drawbacks for predicting crop growth and yield at a regional scale [5–9].

Crop growth models have been developed to simulate crop growth and development, and physiological processes according to environment components at the canopy scale since the mid-1960s [10–11], and advanced computer technology allows simulations close to actual crop growth, which is regulated by the complex interaction of many factors [12]. Despite the noticeable improvement in crop growth model performance, regional prediction of crop growth and yield using crop growth models remains challenging due to the difficulty of obtaining many input parameters of the model at a regional scale and uncertainties in the parameters due to spatial variability [5,7–8].

Remote sensing data provide information related to crop growth status [13–14], and various state variables associated with crop growth have been estimated using vegetation indices derived from remote sensing data. For example, leaf area index (LAI) is estimated with vegetation indices such as the simple ratio index, normalized difference vegetation index (NDVI), and triangular vegetation index [15], and biomass was estimated using NDVI [16]. Although remote sensing data provide spatial information for a specific region [17], the data are not consecutive due to temporal characteristics and atmospheric effects [6,9]. Remote sensing data only show symptoms; they cannot explain the cause of the spectral expression of a crop [18].

These constraints inherent in crop growth modeling and remote sensing can be overcome by integrating remote sensing data into a crop growth model [19–20]; “forcing”, “calibration”, and “updating” strategies have been used to perform this integration [21–23]. The forcing strategy involves replacing state variables derived from remote sensing data into a crop growth model [3]. The state variables derived from remote sensing data are interpolated to obtain daily time series data due to the temporal characteristic of remote sensing data and atmospheric effects [24]. Delécolle and Guérif [25] estimated wheat yield by replacing interpolated LAI derived from Satellite Pour l’Observation de la Terre (SPOT) /High Resolution Visible (HRV) into the Agricultural and Food Research Council (AFRC)-Wheat model. Bouman [26] estimated biomass of winter wheat at harvest by replacing the LAI derived from radar remote sensing into the Simple and Universal Crop Growth Simulator (SUCROS) model. Although the forcing strategy is simple, the initial conditions and/or parameters of the crop growth model should be estimated to improve prediction performance [24]. The calibration strategy is adjusted the initial conditions or the state parameters of the crop growth model using remote sensing data [4]. Fang et al. [27] estimated corn yield in a study region located in the state of Indiana, USA by assimilating LAI derived from MODIS into the CERES-Maize model and adjusted planting date, population, row spacing, and quantity of nitrogen fertilizer by minimizing the difference between simulated LAI and MODIS-derived LAI. Although this method is improved performance of crop yield prediction, it requires the high computational cost to predict crop yield at a large scale because of the repetitive process employed to find the optimum value by minimizing the difference between remote sensing-derived values and simulated values by the crop growth model [27–28]. The updating strategy is updated the state variables whenever remote sensing data is available [22–23]. The ensemble Kalman filter (EnKF), a representative updating method, has been widely used to predict crop yield by assimilating remote sensing data into crop growth models [1–2,29–32]. For example, Li et al. [1] assimilated LAI retrieved from Enhanced Thematic Mapper Plus (ETM+) data into a hydrology crop growth model, which links the World Food Studies (WOFOST) model to better predict corn yields in a study region located in the middle reaches of the Heihe River basin, northwest China; parameters related to maintenance respiration, rooting depth, and soil hydraulic properties were adjusted using EnKF. Wu et al. [31] used EnKF to assimilate MODIS-LAI into the WOFOST model to estimate winter wheat yield in Hengshui district, Hebei Province, China. De Wit and Van Diepen [29] used EnKF to assimilate satellite-derived soil water index into the WOFOST model to estimate winter wheat and maize yield for the period 1992–2000 in Spain, France, Italy, and Germany. This method requires also the most expensive computation cost due to calculating posterior probability density function of the model states [33–34] and the prediction precision using this method varies according to the ensemble size [35].

The objectives of this study were to develop a simple strategy for assimilating MODIS data into a crop growth model using minimum inputs and to evaluate the regional crop yield prediction performance of a simple strategy in a major corn production region, Illinois, USA. In addition, this study focused on examining the possibility of early crop yield predictions using a simple strategy before harvest.

## Materials and methods

### Study area

**Fig 1:**
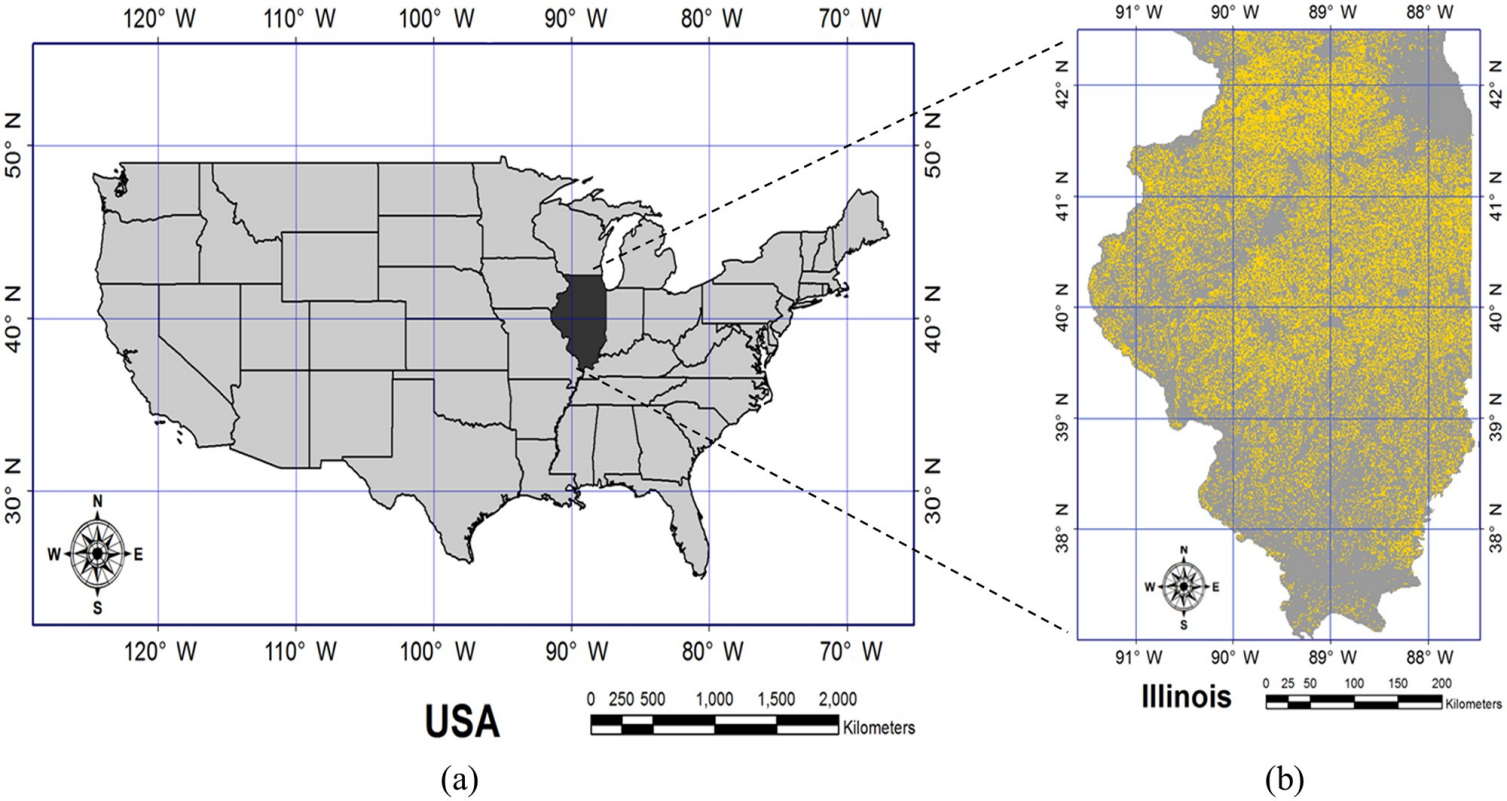
Map of USA showing the location of Illinois (a) and corn crop cover data for Illinois in 2013 (b) (Corn is indicated by yellow) [38].

Illinois (Fig 1a), USA, was selected as the region of interest because this state belongs to a major corn-belt region, so corn production statistics at the county and agricultural district (AD) levels are easily accessible. In 2013, Illinois occupied about 13% and 15% of the national total corn production area and amount, respectively. The annual mean temperature in Illinois is approximately 11°C, and annual precipitation varies from approximately 800 to 1,200 mm according to location. Growing degree days (GDD) of corn hybrids ranges from 2,200 (northern Illinois) to 2,900°C day (southern Illinois). Corn is planted from mid-April to late June and harvested from early September to late November [36]. The irrigation system in Illinois has increased gradually, rising to approximately 625,000 acres in 2014 [37].

### Data and data processing

#### Corn yield and phenology data

Corn yields from 2000 to 2013 in Illinois were obtained from the National Agricultural Statistics Service (NASS) by AD and state to evaluate the reliability of assimilation strategies for predicting regional corn yields. Planted and harvested area in acres and production in bushels were available at the national, state, and county levels. Corn yields, which are measured in bushel per acre in Illinois, were converted to kilogram per hectare.

Corn phenology data in Illinois, which were provided weekly by AD and state, were obtained from the NASS-Illinois Field Office (IFO), and the phenology data were used to estimate corn planting date using crop phenology prediction model [38]. Those data were available in only five ADs, including the Northwest, Northeast, Central, West, and East districts between 2003 and 2012 because phenology data and ADs were not available for several years.

The median DOY on which a given planting stage reached 50% was calculated using linear interpolation because the planting data by AD were surveyed as the planted proportion by week, and the calculated median DOY parameter was used for comparisons with the estimated dates on which a certain phenological stage occurred in the AD.

#### Weather and soil data

Weather and soil data were generated and obtained to use as input data for the crop growth model.

To examine the possibility of early crop yield predictions, weather data were hind-casted using the Pusan National University coupled general circulation model (PNU CGCM) model and downscaled using the dynamic downscaling method [39], and included daily solar radiation (MJ m^-2^ day^-1^), maximum and minimum temperature (°C), and rainfall (mm) at 10-km spatial resolution from 2000 to 2013 in Illinois.

Soil data were obtained from Web Soil Survey (http://websoilsurvey.sc.egov.usda.gov/App/WebSoilSurvey.aspx) operated by the United States Department of Agriculture, Natural Resources Conservation Service of the USA and the data were produced by the National Cooperative Soil Survey. Representative soils by county were selected based on the map unit symbol, which accounts for the largest area of the county and data of representative soil related to chemical and physical properties were obtained. The soil data were processed using Sbuild program within DSSAT 4.6 for subsequent use in the crop growth model. Variables related to soil water contents (e.g., saturated water content, drained upper limit, lower limit of plant extractable soil water, and root growth factor), which are dependent on physical soil properties, were calculated by soil layer using Sbuild. Soil organic carbon (OC) which is an input variable of DSSAT 4.6, was calculated with soil organic matter (OM) obtained from Web Soil Survey using the following equation [40]:

SoilOC(%)=0.4724×soilOM(%) (1)

#### Crop cover data

Corn crop cover data (Fig 1b) were obtained from cropland data layers used by NASS to identify a region where a given crop was grown (https://www.nass.usda.gov/Research_and_Science/Cropland/SARS1a.php). Crop cover data in Illinois were obtained from 2000 to 2013. The projection of crop cover data was converted to a Universal Transverse Mercator (UTM) projection and World Geodetic System (WGS)-84 coordinates at 1-km spatial resolution using ENVI (Exelis VIS; Exelis Visual Information Solutions, Boulder, CO, USA).

#### Surface reflectance data

The MODIS surface reflectance data (i.e. 8-day composited products MOD09A1 with 500-m spatial resolution) from 2000 to 2013 were obtained from Reverb operated by the National Aeronautics and Space Administration (available at http://modis.gsfc.nasa.gov/). The h10v04, h10v05, h11v04, and h11v05 tiled grid data for Illinois were collected from DOY 89 to 329.

The near-infrared (NIR; band 2) and red (band 1) band of MOD09A1 were converted to estimate LAI for corn growing area. First, ban1 and 2 of all tiled data were mosaicked into a single dataset using interface description language (IDL; ExelisVIS). The mosaicked data were converted to UTM projection at 1-km spatial resolution and WGS-84 geographic latitude and longitude coordinates using IDL, which applies the triangulation wrap and nearest neighbor resampling methods. The converted data were resized to fit the size and georeference of crop cover data by year using FWTools, which is a collection of open-source GIS applications. Lastly, the resized data were extracted by corn grid of crop cover data using MATLAB (MathWorks Inc., Natick, MA, USA).

#### Estimation of LAI

LAI was calculated from MOD09A1 of red and NIR bands according to the equations suggested by Nguy-Robertson et al. [15].

$$\text{LAI}=\left\{ \begin{array}{ll} \left(\text{NDVI}-0.28\right)/0.18, & \text{NDVI}\leq 0.7\\ \left(\text{SR}-1.0\right)/0.35, & \text{NDVI}>0.7 \end{array}\right.$$ (2)

where NDVI [41] and SR [42] represent the normalized difference vegetation index and simple ratio, respectively. NDVI and SR were calculated as follows:

NDVI=(NIR-red)/(NIR+red) (3)

SR=NIR/red (4)

where NIR and red represent the reflectance of near-infrared and red band spectra, respectively.

### Crop growth model

The CERES-Maize model [13] in DSSAT4.6.0.020 [44–45], which has been widely used to simulate maize growth and yield [46], was employed for this study. The CERES-Maize model simulates daily changes in physiological processes (e.g., phenological development, crop growth, biomass partitioning, nutrient uptake, and water use) in response to changes in environmental components (e.g., solar radiation, temperature, and rainfall) and management practices (e.g., planting date and amount of fertilizer) and final yield [46–48]. In this study, CERES-Maize model coded in Fortran was used to modify the code according to assimilation strategies.

### Data assimilation strategies for predicting regional corn yields

Two assimilation strategies were employed for predicting regional corn yields. The first assimilation strategy was to employ planting date and maturity group estimated using MODIS-derived LAD-logistic function for each grid into the CERES-Maize model. The second assimilation strategy was to further employ daily LAI (i.e. LAI_RS_) and water stress factors (i.e. TURFAC_est_ and SWFAC_est_) estimated using the MODIS-derived LAD-logistic function for each grid into the CERES-Maize model.

To simulate the CERES-Maize model, management practices such as planting density, depth, and amount of fertilizer are presented in Table 1. The first and second fertilizers were applied at the planting date and 2 weeks after planting, respectively. Although management practices in corn fields of Illinois were diverse due to a large area, management practices for all grid were applied equally by referring to the information of Nafziger [36], and fertilizer amounts were sufficiently configured to exclude fertilizer stress.

**Table 1:** Management settings for the crop growth model.

Management	Unit	Value
Planting density	plant m^-2^	7.41
Planting depth	cm	4.5
Amount of first fertilizer	kg ha^-1^ (N-P-K)	90-30-69
Amount of second fertilizer	kg ha^-1^ (N-P-K)	90-0-0

The assimilation strategies are employed in all corn growing areas at 1-km grid. The soil and weather data of all grids were decided using ArcMap (Esri, Redlands, CA, USA) due to the difference of grid size in LAI derived from MODIS, soil, and weather data.

#### Assimilation strategy with planting date and maturity group

**Fig 2:**
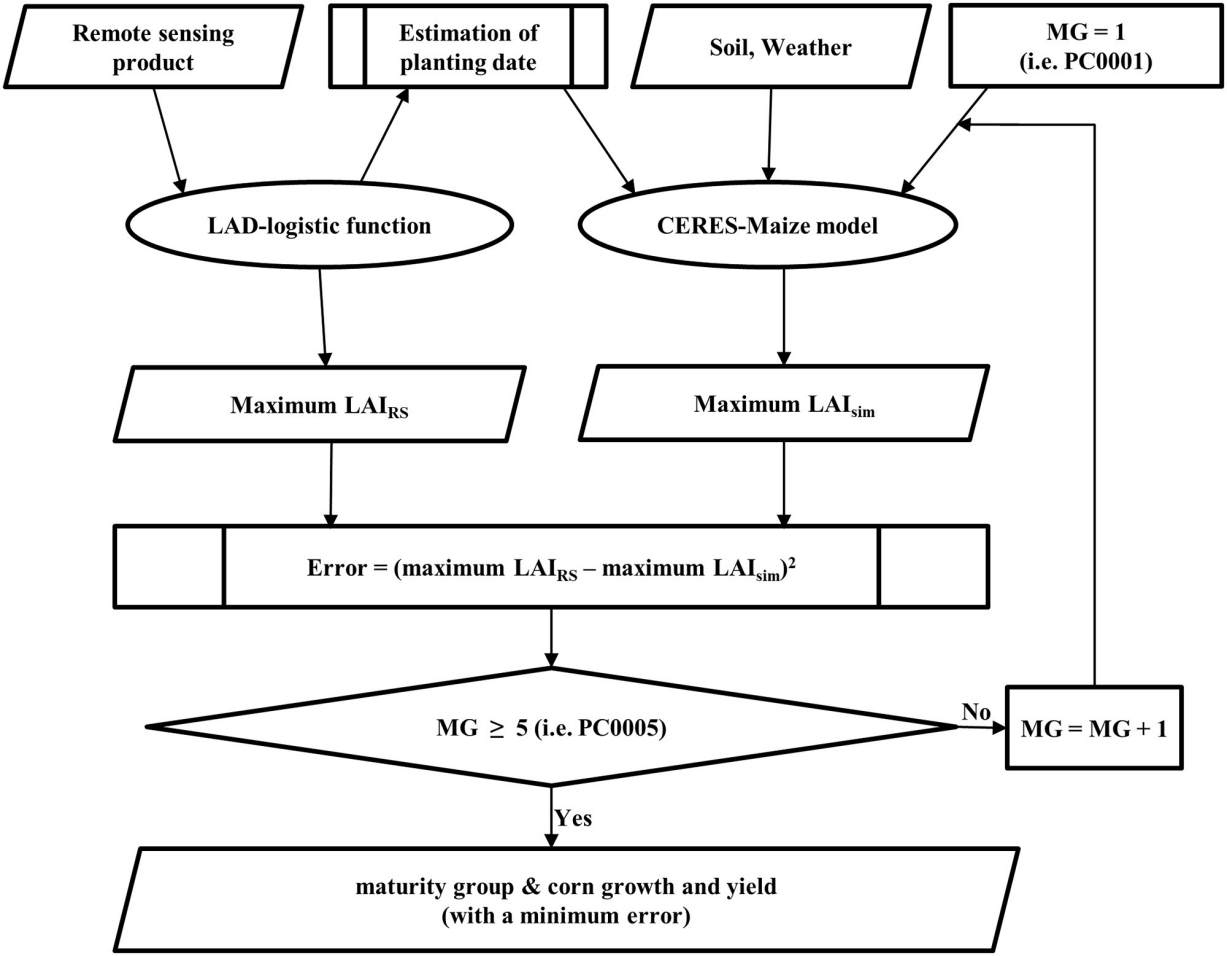
Flowchart for assimilating the estimated planting date and maturity group (RS: Estimated value derived from MODIS data; sim: Simulated by the CERES-Maize model; MG: Maturity group).

Planting date and maturity group estimated using MODIS-derived LAD-logistic function for each grid were assimilated into the CERES-Maize model to predict corn yield for each grid, as shown in Fig 2.

The planting date was estimated using remote sensing data, and the CERES-Maize model coded in Fortran was modified to estimate maturity group. CERES-Maize model was simulated as much as the number of cultivar coefficients to estimate maturity group for a given grid under two water supply conditions (i.e., “rain-fed”: the amount of rainfall in the weather file and “auto-irrigation”: irrigation and water management simulation options are set to automatic when required), maximum LAI values simulated with each cultivar coefficient compared to the maximum LAI value estimated using remote sensing data, and the cultivar coefficient that had the smallest difference was designated as the maturity group for a given grid. Corn yields with the assimilation of estimated planting date and maturity group by grid were simulated under the rain-fed and auto-irrigation conditions, and the corn yields by grid were aggregated to the AD and state levels.

#### Assimilation strategy with additional daily LAI and water stress factors

**Fig 3:**
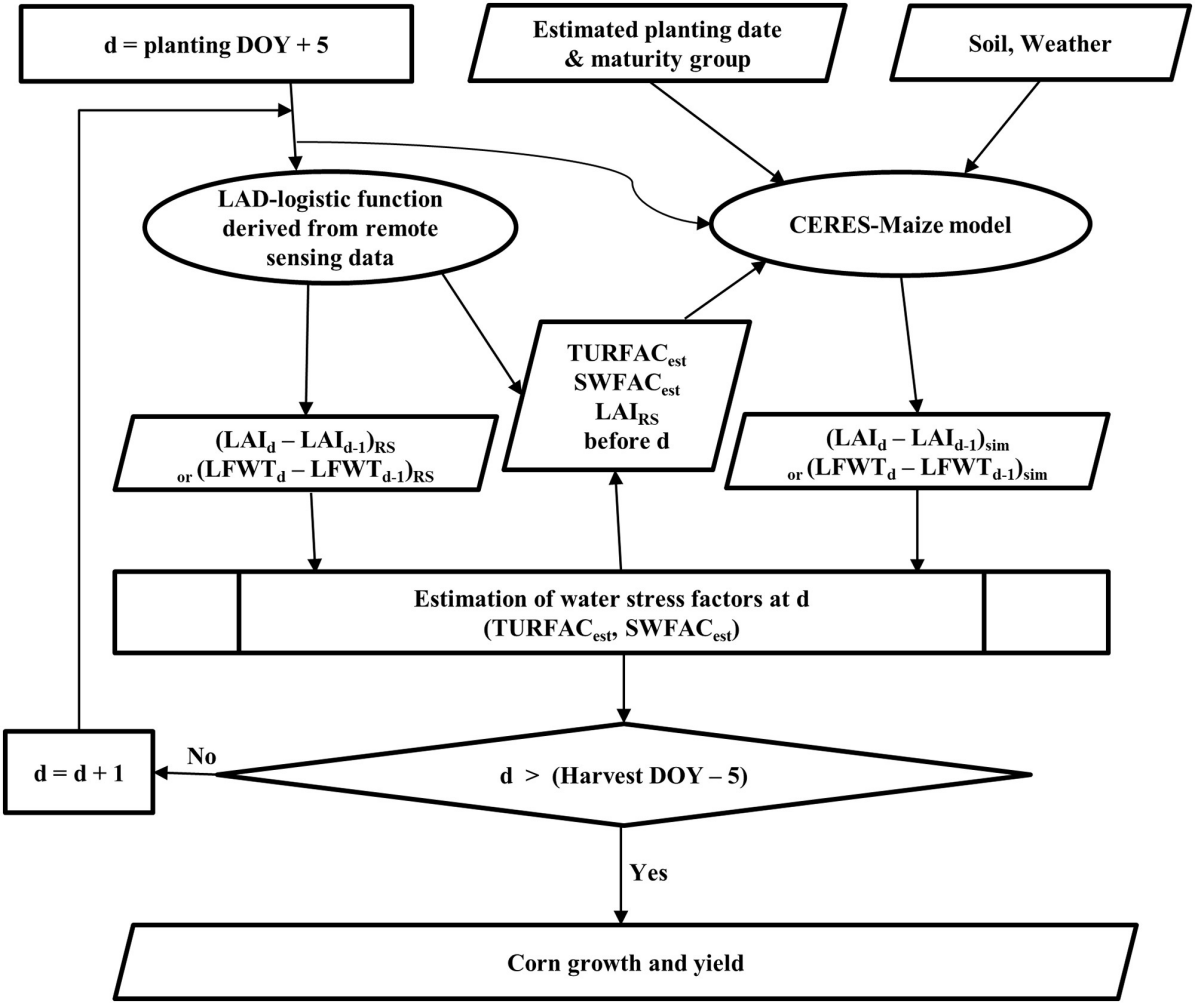
Flowchart for assimilating estimated daily leaf area index (LAI) and water stress factors (i.e. TURFAC and SWFAC) in addition to the estimated planting date and maturity group (RS: Estimated value derived from MODIS data; sim: Simulated by the CERES-Maize model; est: Estimated; DOY: Day of year; LFWT: Leaf weight; d: Current day; d-1: Previous day).

Daily LAI and water stress factors estimated using the MODIS-derived LAD-logistic function for each grid were additionally assimilated into the CERES-Maize model to predict corn yields by a grid, as shown in Fig 3.

The estimated planting date and maturity group were used for the CERES-Maize model simulation and the model coded in Fortran was modified to estimate daily water stress factors. The water stress factors on a given day were estimated using growth rate estimated from MODIS-derived LAD-logistic function to that simulated by the CERES-Maize model on a given day, and all daily LAI and water stress factors estimated before a given day were used again as input of CERES-Maize model to estimate water stress factors on a given day. The CERES-Maize model was simulated as much as the number of a growth period to estimate daily water stress factors during a growth period because CERES-Maize model is a daily time-step simulation model. The corn yields by grid were aggregated to the AD and state levels.

### Estimation of assimilation data

#### Estimate of planting date

The planting date was estimated via the crop phenology prediction model [38] using a logistic function describing the seasonal changes in LAD which is the integrated LAI value for a specific period. A logistic function was used to represent the integral of seasonal changes in LAI (Eq 4) over time as follows:

$$LAD(t)=\int LAI(t)dt=\frac{b_{3}}{1.0+exp[-b_{1}(t-b_{2})]}$$ (5)

where t indicates days after planting and b1, b2, b3 represent the LAI growth rate, the date of the maximum LAI, and the cumulative LAI at physiological maturity, respectively. Ban et al. [38] established a crop phenology model (Eq 6) using the parameters (b_1_ and b_2_) of the LAD-logistic equation as predictor variables. The data of phonological stage (D) was estimated as follows:

$$D=b_{2}+\tau +\rho /b_{1}$$ (6)

Where τ represents the difference between the date when LAI reaches the maximum value and the date of a given phenological stage, ρ represents the effect of an increase in LAI on phenological change over the growing season, and b_1_ and b_2_ represent the rate of LAI growth and the date when the LAI value reaches the maximum, respectively. The τ and ρ values for planting date by the end of the DOY (EOD) were estimated with calibration datasets of Ban et al. [38] using the phenology data reported by NASS (Table 2).

**Table 2:** Estimated parameters for the crop phenology prediction model for planting date.

EOD	*τ*	*ρ*
209	-4.73	0.96
257	-10.74	0.06
321	-8.77	0.36

EOD denotes the last date of remote sensing data products used to fit the logistic function and sets to verify the possibility of early crop yield predictions using a simple strategy before harvest. EODs 209, 257, and 321 were selected to evaluate corn yield predictions, and EODs 209, 257, and 321 are near the usual corn flowering DOY in Illinois, the earliest DOY when the LAD-logistic function could be established reliably, and the date on which the corn harvest was completed, respectively. RMSE and normalized RMSE (NRMSE) for validation datasets of 209, 257, and 321 EOD were 7.5 days and 6.1%, 6.9 days and 5.6%, and 7.3 days and 5.9%, respectively.

#### Estimate of daily LAI and leaf weight (LFWT)

Daily LAI was estimated by differentiating the LAD-logistic equation (Eq 5) in terms of time, and the parameters (b_1,_ b_2,_ and b_3_) of the LAD-logistic equation were used.

$${LAI}_{RS}=\frac{b_{3}∙b_{1}∙exp[-b_{1}(d-b_{2})]}{{\left\{1.0+exp[-b_{1}(d-b_{2})]\right\}}^{2}}$$ (7)

where b_1_, b_2_, and b_3_ represent the rate of LAI growth, the date with maximum LAI, and the cumulative LAI at physiological maturity, respectively, and d indicates a given date. Daily LFWT was calculated using LFWT computation equation of CERES-Maize model [43–45].

$${LFWT}_{RS}={\left({LAI}_{RS}/PLTPOP/0.0001/267.0\right)}^{1.25}$$ (8)

where PLTPOP is plant population (plants m^-2^).

#### Estimate of corn maturity group

The CERES-Maize model was simulated to estimate corn maturity group by grid under the rain-fed and auto-irrigation simulations. Corn maturity has been classified by the number of days from planting to harvest and is mainly determined by GDD which is accumulated from planting to maturity [49]. Illinois has various ranges of GDD due to a large area. Thus, corn cultivars of various maturities have been cultivated in Illinois [36]. Although genetic characteristics are different between corn cultivars belonging to the same maturity group, this paper assumed that cultivars belonging to the same maturity group have the same genetic characteristics, and the genetic coefficients of corn hybrids included in DSSAT 4.6 were used to estimate corn maturity for each grid.

The cultivar coefficients for five generic corn hybrids, identified as PC0001–PC0005 according to GDD and included in DSSAT 4.6 (Table 3), were used to identify the maturity group of the corn cultivar in a given grid. The RMSE between the maximum LAI value estimated by the LAD-logistic function (i.e. LAD(b_2_)-LAD(b_2_-1) in Eq 5) and that simulated by the CERES-Maize model with each maturity group during the growing season was calculated by maturity groups (Table 3), and the maturity group that had the smallest RMSE was designated as the mature cultivar for a given grid.

**Table 3:** Genetic coefficients used to estimate corn maturity groups.

VAR#	VRNAME	P1	P2	P5	G2	G3	PHINT
PC0001	2500–2600 GDD	160.0	0.75	780.0	750.0	8.5	49.0
PC0002	2600–2650 GDD	185.0	0.75	850.0	800.0	8.5	49.0
PC0003	2650–2700 GDD	212.0	0.75	850.0	800.0	8.5	49.0
PC0004	2700–2750 GDD	240.0	0.75	850.0	800.0	8.5	49.0
PC0005	2750–2800 GDD	260.0	0.75	850.0	800.0	8.5	49.0

VAR#: Identification code or number for a specific cultivar, VRNAME: Name of cultivar, P1: Thermal time from seedling emergence to the end of the juvenile phase in degree day, P2: Photoperiod sensitivity (0–1.0) expressed in days delayed for each hour increase in photoperiod above the longest photoperiod (12.5 hours) at which development proceeds at a maximum rate, P5: Thermal time from silking to physiological maturity in degree days, G2: Potential kernel number in no. per plant, G3: Potential kernel filling rate during the linear grain filling stage in mg/kernel/day, PHINT: interval between leaf tip appearances in degree.

#### Estimating daily water stress factors

The most crucial limitation for crop model-based crop yield prediction in regions where rain-fed and irrigated areas are mixed, as in Illinois, is to assess water stress as a critical factor for crop growth and yield. Leaf growth is very sensitive to inhibition by water stress [50], and leaf area growth rate is a good indicator of water stress. The water stress factors (i.e., TURFAC and SWFAC) in the CERES-Maize model were estimated using the MODIS-derived LAD-logistic function. TURFAC and SWFAC variables, which are water stress factors for leaf area expansion and soil water stress effect on photosynthesis, respectively, have values ranging from 0.0 to 1.0 [51]. In the CERES-Maize model, these variables are calculated as the ratio of total root water uptake to potential transpiration, and if the ratio is less than a specific value, the variables have values < 1.0 [52]. The TURFAC and SWFAC variables affect the rates of crop growth and development (e.g., leaf expansion and senescence) [53]. Finally, crop yields decrease in response to these variables [54]. In the CERES-Maize model, TURFAC and SWFAC variables can be estimated using daily leaf growth rate related to leaf growth and were estimated by the variable differently for each crop growth stage (i.e. ISTAGE, Table 4, [55]) because the growth variables related to water stress factor are different.

**Table 4:** Description of ISTAGE variable in CERES-Maize model.

ISTAGE	Description
1	Emergence to end of juvenile stage
2	End of juvenile stage to tassel initiation
3	Tassel initiation to end of leaf growth
4	End of leaf growth to beginning effective grain filling period
5	Beginning to end of effective grain filling period
6	End of effective grain filling period to physiological maturity

LAI in CERES-Maize model [43–45] is computed as follows:

LAI=(PLA-SENLA)∙PLTPOP∙0.0001 (9)

where PLA is plant leaf area (cm^2^ plant^-1^), SENLA is normal leaf senescence today (cm^2^ plant^-1^), and PLTPOP is plant population (plants m^-2^). The PLA was differently computed by ISTAGE

In ISTAGE 1 and 2, PLA is computed as follows:

PLA=PLA+PLAG (10)

where PLA is plant leaf area (cm^2^ plant^-1^), PLAG is leaf area growth rate (cm^2^ plant^-1^ day^-1^), and is computed as follows:

PLAG=3.0∙XN∙XN∙TI∙AMIN1(TURFAC,(1.0-SATFAC),PSTRES2,KSTRES) (11)

where XN is number of oldest expanding leaf, TI is fraction of a phyllochron interval which occurred as a fraction of today’s daily thermal time, AMIN1 is function which selects a minimum value, TURFAC is soil water stress effect on expansion, SATFAC is water logging stress factor, and indicate growth rate per a day, PSTRES2 and KSATRES are phosphorus and potassium stress factor, respectively. In this study, it was assumed that PLAG is only affected by water stress factor, TURFAC. Then, TURFAC can be estimated by the ratio of actual to potential daily leaf area growth rate.

In ISTAGE 3, PLA is computed as follows:

$$PLA={\left(LFWT+GROLF\right)}^{0.8}∙267.0$$ (12)

where LFWT is leaf weight (g plant^-1^), and GROLF is leaf weight growth rate (g plant^-1^ day^-1^). GROLF is computed as follows:

$$GROLF=0.00116∙PLAG∙{\left(PLA\right)}^{0.25}$$ (13)

where PLAG is leaf area growth rate and PLA is plant leaf area. GROLF is also affected by TURFAC as PLAG is affected by it. Thus, TURFAC can be estimated with daily leaf weight growth rate, and the daily leaf weight was calculated with daily leaf area growth. STAGE 4, 5 and 6 is senescence phase, LAI is depended on SENLA (i.e. normal leaf senescence today (cm^2^ plant^-1^)). SENLA is computed as follows:

SENLA=SENLA+PLAS (14)

where PLAS is the rate of senescence of leaf area on one plant (cm^2^ day^-1^). PLAS computed as follows:

PLAS=(PLA-SENLA)∙(1.0-AMIN1(SLFW,SLFC,SLFT,SLFN,SLFP) (15)

where PLA is plant leaf area (cm^2^ plant^-1^), SENLA is normal leaf senescence today (cm^2^ plant^-1^), SLFW is leaf senescence factor due to water stress, SLFC is leaf senescence factor due to competition for light, SLFT is leaf senescence factor due to temperature, SLFN is leaf senescence factor due to nitrogen stress, and SLFP is leaf senescence factor due to phosphorus stress. This paper assumed that plant is only affected by water stress, and SLFW related to water stress is computed as follows:

SLFW=(1-FSLFW)+FSLFW∙SWFAC (16)

where FSLFW is fraction of leaf area senesced due to 100% water stress (1 day^-1^), and SWFAC is soil water stress effect on growth. FSLFW is set to 0.05 in CERES-Maize model. Thus, SWFAC can be estimated with daily leaf area senescence rate. If one water stress factor can be estimated by each ISTAGE, the other water stress factor can be also estimated. TURFAC was computed as follows:

$$TURFAC=\left(\frac{1}{RWUEP1}\right)∙\frac{TRWUP}{EP1},if\frac{TRWUP}{EP1}<RWUEP1$$ (17)

where RWUEP1 is factor to modify water stress for cell expansion (mm day^-1^), TRWUP is total root water uptake (mm day^-1^), and EP1 is potential plant transpiration (mm day^-1^). SWFAC was computed as follows:

$$SWFAC=\frac{TRWUP}{EP1},if\;EP1<TRWUP$$ (18)

SWFAC was also calculated as follows using Eqs 16 and 17:

SWFAC=TURFAC∙RWUEP1 (19)

In CERES-Maize model, RWUEP1 in TURFAC equation is set to 1.5. Therefore, if one water stress factor can be estimated, the other water stress factor can be also estimated. The water stress factors below 0.0 were set to 0.0, and above 1.0 were set to 1.0.

In ISTAGE 1 and 2, TURFAC is estimated as follows:

$${TURFAC}_{est}={\left({LAI}_{d}-{LAI}_{d-1}\right)}_{RS}/{\left({LAI}_{d}-{LAI}_{d-1}\right)}_{sim}*{TURFAC}_{sim}$$ (20)

where LAI is leaf area index (m^2^ m^-2^), RS is estimated value derived from MODIS data, sim is simulated value of CERES-Maize model, d is current day, and d-1 is previous day.

In ISTAGE 3, TURFAC is estimated as follows:

$${TURFAC}_{est}={\left({LFWT}_{d}-{LFWT}_{d-1}\right)}_{RS}/{\left({LFWT}_{d}-{LFWT}_{d-1}\right)}_{sim}*{TURFAC}_{sim}$$ (21)

where LFWT is leaf weight (g plant^-1^).

In ISTAGE 1–3, SWFAC is estimated as follows:

$${SWFAC}_{est}={TURFAC}_{est}*1.5$$ (22)

In ISTAGE 4–6, SWFAC and TURFAC are estimated as follows:

$${SWFAC}_{est}=1.0-(-{\left({LAI}_{d}-{LAI}_{d-1}\right)}_{RS}/PLAS*(1.0-{SWFAC}_{sim}))$$ (23)

$${TURFAC}_{est}={SWFAC}_{est}/1.5$$ (24)

Water stress factors were calculated from 5 days after planting to 5 days before harvest and these days were considered the emergence date and physiological maturity date, respectively. The daily water stress factors and LAI estimated using the MODIS-derived LAD-logistic function were integrated into the CERES-Maize model for predicting corn growth and yield. In CERES-Maize model, the corn yield (kg ha^-1^) is computed as follows:

YIELD=GRNWT∙10.0∙EARS (25)

where GRNWT is grain weight (g plant^-1^), and EARS is ears per m^2^. GRNWT is computed as follows:

GRNWT=GRNWT+GROGRN (26)

where GROGRN is a daily growth of the grain rate (g day^-1^). GROGRN is computed as follows:

GROGRN=RGFILL∙GPP∙G3∙0.001∙(0.45+0.55∙SWFAC) (27)

where RGFILL is a rate of grain fill (mg day^-1^) as affected by temperature on relative grain filling rate, GPP is grain number per plant (grains plant^-1^), and G3 is potential kernel growth rate (mg kernel^-1^ day^-1^). Daily growth of the grain (i.e., GROGRN) is affected by SWFAC during grain filling period. Finally, corn yield is affected by SWFAC.

### Degree of agreement analysis

Three types of statistics, namely R^2^, RMSE, and NRMSE, were determined for crop yields. Corn yield for each grid was summarized by individual season and AD/state to compare with the reported yields at the regional scale. Corn yields were also aggregated to compare the yields predicted with those reported in Illinois by season. The RMSE value was determined as follows:

$$RMSE=\sqrt{\frac{1}{n}\sum_{i=1}^{n}{\left(P_{i}-O_{i}\right)}^{2}}$$ (28)

where n represents the number of comparisons, and P_i_ and O_i_ are estimated and reported data, respectively. The NRMSE was determined as follows [56]:

$$NRMSE=RMSE\times \frac{100}{M}$$ (29)

where M is the mean reported yield. Depending on the NRMSE value, the predicted results are considered excellent (NRMSE < 10%), good (10% < NRMSE < 20%), fair (20% < NRMSE < 30%), and poor (NRMSE > 30%).

## Results

### Corn yields at the AD level

**Fig 4:**
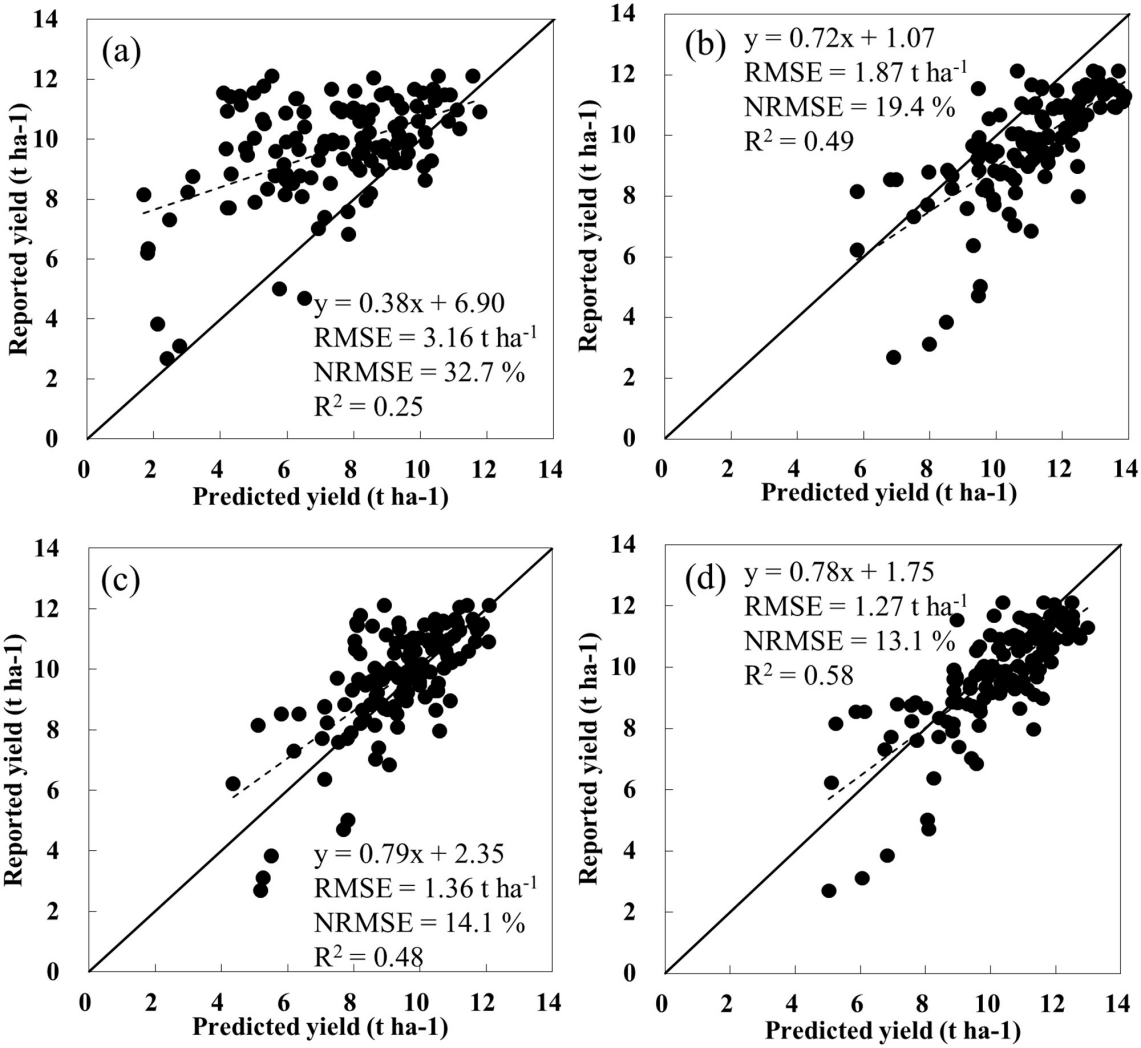
Comparison of reported and predicted corn yields at the agricultural district (AD) level with different data assimilation and simulation conditions from 2000 to 2013 in Illinois, USA, at end of day of year (DOY) [EOD] 321 [The CERES-Maize model was used for the simulation, with estimated planting date and maturity group under the (a) rain-fed and (b) auto-irrigation conditions, and simulated by assimilating the MODIS-derived daily leaf area index (LAI) and water stress factors in addition to estimated planting date and maturity group under the (c) rain-fed and (d) auto-irrigation conditions].

As presented in Fig 4, corn yields simulated under different irrigation options and MODIS-derived data assimilation mehtods were compared with reported corn yields. Corn yields at the AD level were simulated under rain-fed and auto-irrigation conditions using the CERES-Maize model, which was assimilated with planting date and maturity group estimated from the LAD-logistic function. In addition to the estimated planting date and maturity group, the estimated daily LAI and water stress factors were assimilated for predicting corn yields at the AD level under the rain-fed and auto-irrigation conditions.

The simulation using the estimated planting date and maturity group under the rain-fed condition tended to underestimate corn yield and showed very poor performance (Fig 4a), whereas the simulation involving the same assimilation of the estimated planting date and maturity group under the auto-irrigation condition tended to overestimate corn yield, but the prediction performance was improved compared to that under the rain-fed condition (Fig 4b). In addition, further assimilation of daily LAI and water stress factors improved the prediction performance of corn yield under both rain-fed and auto-irrigation conditions (Fig 4c and 4d), and the simulation by further assimilating daily LAI and water stress factors under the auto-irrigation condition showed the best performance (Fig 4d).

### Corn yields at the state level

**Fig 5:**
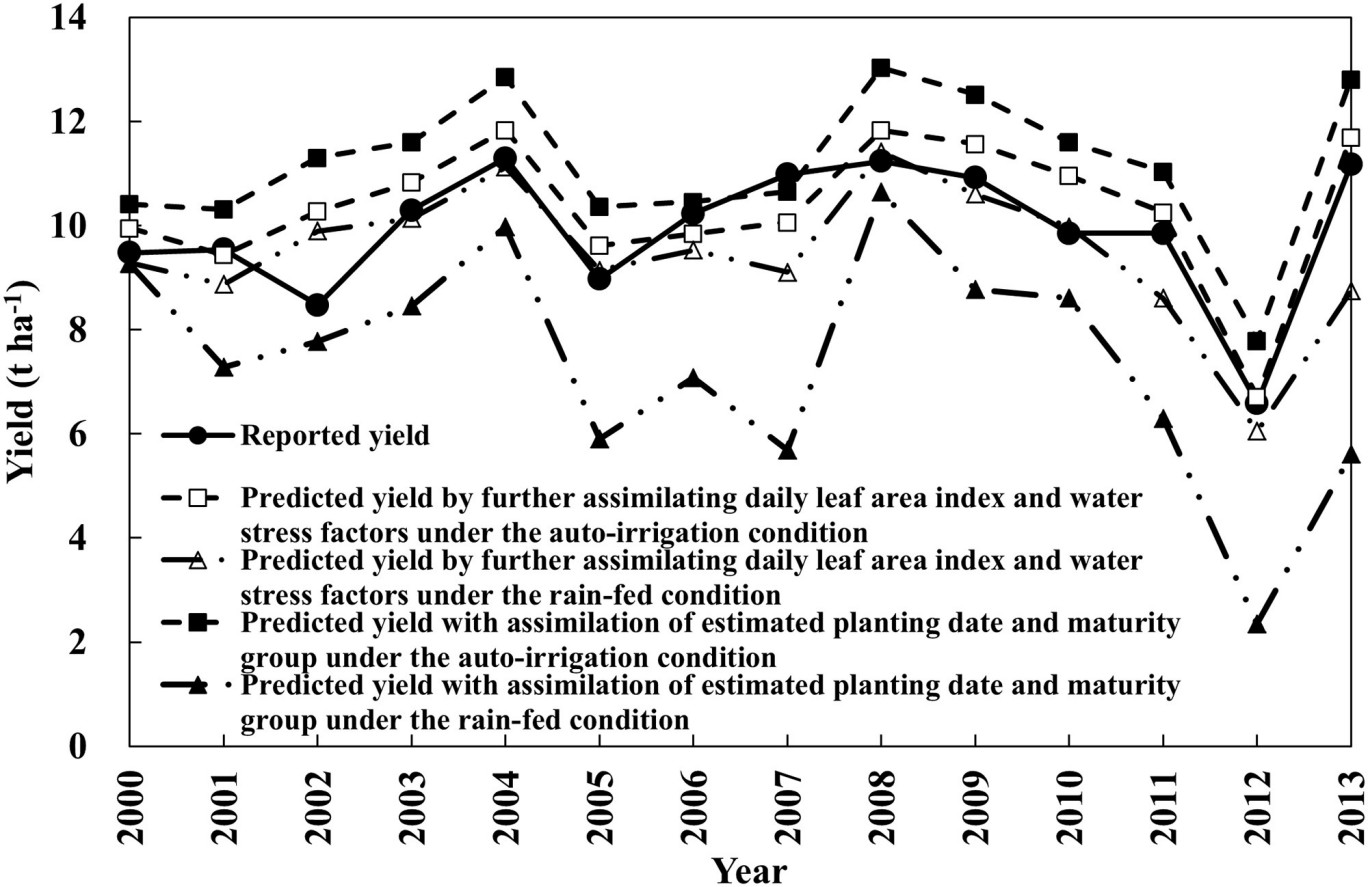
Reported and predicted corn yields at the state level with different data assimilation and simulation conditions from 2000 to 2013 in Illinois, USA, at end of day of year (DOY) [EOD] 321.

Corn yields predicted at the AD level were aggregated for comparison with the reported corn yields at the state level, as shown in Fig 5. The overall results were similar to the predicted corn yields at the AD level. Yearly corn yields simulated with the estimated planting date and maturity group under the rain-fed condition were much lower than the reported corn yields and poorly represented the yearly variations in corn yield at the state level, whereas yearly corn yields simulated under the auto-irrigation condition were higher than the reported corn yields and represented the yearly variation in corn yield fairly well. In each simulation condition, further assimilation of daily LAI and water stress factors with the estimated planting date and maturity group improved simulation performance by predicting corn yield and representing the yearly yield variation better than the simulation without additional assimilation of daily LAI and water stress factors was able to do. Predicted corn yields by further assimilating daily LAI and water stress factors under the auto-irrigation condition were closest to the reported corn yields.

The statistical indices for the corn yields predictions at the state level are shown in Table 5. The corn yield simulation using the estimated planting date and maturity group under the rain-fed condition showed the worst performance for all EODs, whereas the corn yield simulation with the same assimilation under the auto-irrigation condition showed much better performance, increasing the R^2^ value from 0.38 to 0.72 and decreasing the RMSE from 3.07 to 1.47 t ha^-1^ at EOD 321. Additional assimilation of daily LAI and water stress factors to the estimated planting date and maturity group under both simulation conditions also resulted in further improvement of the corn yield prediction. Additional assimilation under the rain-fed condition increased the R^2^ value from 0.38 to 0.55 and decreased the RMSE from 3.0 to 1.02 t ha^-1^ for the EOD 321 simulation, and additional assimilation under the auto-irrigation condition increased the R^2^ value from 0.72 to 0.78 and decreased the RMSE from 1.47 to 0.75 t ha^-1^ for the EOD 321 simulation. The simulation by further assimilating daily LAI and water stress factors under the auto-irrigation condition showed the best performance. Although corn yield simulation with the additional assimilation of daily LAI and water stress factors at EOD 209 was worse than those for the other EODs, the level of agreement statistics for all EODs showed good performance, and performance improved with increasing EOD. The R^2^, RMSE, and NRMSE values for predicting corn yields at all EODs were >0.57, <0.91 t ha^-1^, and 9.19%, respectively.

**Table 5:** Statistical indices for predicted corn yields at the state level with different data assimilation and simulation conditions by end of day of year (DOY) [EOD].

Assimilation method	R^2^			RMSE (tha^-1^)			NRMSE (%)		
	EOD 209	EOD 257	EOD 321	EOD 209	EOD 257	EOD 321	EOD 209	EOD 257	EOD 321
Default_rain	0.37	0.33	0.38	2.78	2.93	3.00	28.05	29.50	30.26
Default_auto	0.73	0.71	0.72	1.60	1.53	1.47	16.15	15.42	14.79
Stress_rain	0.34	0.59	0.55	1.69	0.86	1.02	16.99	8.66	10.26
Stress_auto	0.57	0.78	0.78	0.91	0.88	0.75	9.19	8.91	7.58

Default_rain: Corn yield prediction with assimilation of estimated planting date and maturity group under the rain-fed condition, Default_auto: Corn yield prediction with assimilation of estimated planting date and maturity group under the auto-irrigation condition,.Stress_rain: Corn yield prediction with additional assimilation of daily leaf area index and water stress factors under the rain-fed condition, Stress_auto: Corn yield prediction with additional assimilation of daily leaf area index and water stress factors under the auto- irrigation condition.

## Discussion

Regional crop yield predictions using a crop growth model are challenging due to the large uncertainty inherent in the input data and parameters (e.g., soil properties, initial condition, crop parameters, weather, and management practices) [57]. Although remote sensing data provide information related to crop growth status at a regional scale, the data are not consecutive. These constraints can be overcome by assimilating remote sensing data into a crop growth model [58]. Three strategies (i.e., forcing, calibration, and updating) were used to integrate remote sensing data into crop growth models, and prediction performance improved through the use of these strategies. However, crop growth and yield predictions using these strategies were spatially limited due to estimates of the initial conditions and/or parameters for the crop growth model using a calibration dataset and required a high computation cost due to a large ensemble size in order to reduce the errors [35].

In this study, a simple data assimilation strategy was developed to improve regional corn yield prediction performance by integrating information on crop management and growth derived from MODIS data into the CERES-Maize model using minimum inputs. This method does not need to estimate the initial conditions and/or parameters of CERES-Maize model. Only planting date, maturity group, daily LAI, and water stress factors, which were estimated using a MODIS-derived LAD-logistic function, were assimilated into the CERES-Maize model to improve accuracy for predicting corn yield. The corn yield simulation at the AD and state levels using only the estimated planting date and maturity group showed under estimation under the rain-fed condition, whereas overestimation was observed under the auto-irrigation condition (Figs 4 and 5 and Table 5). This result suggests that maize has been grown under partial irrigation situations in Illinois. Bridges et al. [37] reported that irrigation systems have increased gradually in Illinois, rising to approximately 625,000 acres in 2014. It is most important to estimate the temporal and spatial variations of water stress directly using remote sensing and consider water stress when simulating crop growth and yield in order to improve the corn yield prediction in a region such as Illinois, where irrigation is only practiced partially and rainfall is insufficient during the growing season. Water is one of the most important factors limiting crop growth and yield [59–61]. Leaf growth is reduced, dry matter allocation to the root is increased, and the root-to-shoot ratio decreases when water stress occurs in a plant [62–64]. Therefore, leaf growth rate is a good criterion to use in assessing the degree of water stress, and water stress factors can be estimated using daily crop growth rate based on the balance between soil water supply and crop water demand [46]. Daily water stress factors employed in the CERES-Maize model were estimated by the ratio of daily leaf area/weight growth rate estimated from the LAD-logistic function to the daily leaf area/weight growth rate estimated by the CERES-Maize model under the auto-irrigation and rain-fed conditions using Eqs 20–24. In addition to the estimated planting date and maturity group, the additional assimilation of MODIS-derived daily LAI and water stress factors into the CERES-Maize model further improved yield prediction performance under both irrigation conditions. However, the further assimilation showed much better performance under the auto-irrigation condition than under rain-fed-condition (Figs 4 and 5 and Table 5), showing the R^2^ value of 0.78 and RMSE of 0.75 t ha^-1^ for the state corn yield prediction at EOD 321. This result would be ascribable to the followings: Though the CERES-Maize model calculates the actual growth rates by multiplying the potential growth rate and the water stress factors ranging from 0.0 to 1.0 together [43–44], the potential growth rates would be underestimated and the stress factors be overestimated in return under “rain-fed” condition as compared to those under “auto-irrigation” condition. However, the corn yield simulation with the additional assimilation of daily LAI and water stress factors showed slightly poorer performance at EOD 209 than at EOD 257 and EOD 321. This may have been caused by the unreliable estimate of daily LAI and water stress factors, which was calculated from the estimated daily LAI. Ban et al. [38] reported that the MODIS-derived LAD-logistic function parameters may not have been estimated reliably at the EOD before the date of maximum daily LAI, resulting in an unreliable estimate of daily LAI.

The RMSE value at EOD 321 for the state-level corn yields predicted with daily LAI and water stress factors estimated using the MODIS-derived LAD-logistic function was 0.75 t ha^-1^. Ines et al. [30] used predicted corn yields from 2003 to 2009 in Story County, Iowa, USA with a RMSE value of 1.4 t ha^-1^ using EnKF to assimilate soil moisture and/or MODIS-LAI into the CERES-Maize model, and Fang et al. [27] predicted corn yield in several counties in Indiana, USA, with an RMSE value of 0.85 t ha^-1^ using the Markov model. Although the region and scale in the current study differed from those in previous studies, the RMSE value of the predicted corn yields achieved by additional assimilation was smaller than the RMSE values reported in the previous studies. In addition, the RMSE value at a fairly early stage of EOD 257 was 0.88 t ha^-1^, being similar to those for the previous studies. The first previous study used a recursive process that calculated the posterior probability density function using EnKF, and the second previous study also used a repetitive process that adjusted the environmental conditions and parameters of the crop growth model by minimizing the difference between remote sensing-derived values and simulated values in the crop growth model. For example, Fang et al. [27] estimated planting date, population, row spacing, and quantity of nitrogen fertilizer by minimizing the difference between simulated LAI and MODIS-derived LAI. These methods require a high computational cost to predict regional crop growth and yield and a large input dataset, as well as local characteristics for the estimated parameters in the crop growth model, and would be spatially limited. However, the present assimilation strategy using minimum data (i.e., daily water stress factors, daily LAI, planting date, and maturity group) required only a repetitive process to estimate water stress factors of CERES-Maize model and used only a few input parameters.

By assimilating daily LAI and water stress factors, the predicted yearly trend in the state corn yields was very close to the reported trend of yearly corn yields. However, corn yields were predicted to be much higher than the reported corn yield in 2002 and 2010 (Fig 5), indicating that factors other than water stress decreased corn yields in those years. Actual yields (i.e., reported yields) are largely affected by regional socioeconomic conditions, crop management, and disease (e.g., fertilizer and biocide use) [65–66]. Although remote sensing data were used to overcome the uncertainties caused by the large scale, not all of the information about the actual yield loss was addressed. The accuracy of corn yield predictions would improve by adding reliable information about other components (e.g., insects, pests, and extreme weather events).

## Conclusion

A simple approach to predict regional corn yield was developed by assimilating MODIS product data into the CERES-Maize model using minimum inputs. This method requires only a repetitive process to estimate water stress factors of the CERES-Maize model. Minimum inputs comprising planting date, fertilizer amount, genetic coefficients, soil, and weather data were used to simulate corn growth and yield using CERES-Maize model. Planting date, corn maturity group, daily LAI, and daily water stress factors estimated using the MODIS-derived LAD-logistic function were directly assimilated into the CERES-Maize model to predict regional corn yield in Illinois, USA. The corn yield predictions using only estimated planting date and maturity group performed very poorly under the rain-fed condition at both the AD and state levels, whereas corn yield prediction performance improved by simulation under the auto-irrigation condition. Moreover, adding the daily LAI and water stress factors into the MODIS-derived LAD-logistic function further improved corn yield prediction performance under the both rain-fed and auto-irrigation conditions. However, the simulation by further assimilating daily LAI and water stress factors under the auto-irrigation condition showed the best performance. In addition, earlier corn yield prediction at DOY 257 was possible without degrading accuracy. This simple approach was successful for predicting regional corn yield with considerable accuracy and precision in Illinois, USA. However, this method needs to be examined in regions with more diverse agro-climatic and agro-technology conditions.
